# Supplementary material for: Novel Insights on the Toxicity of Phycotoxins on the Gut through the Targeting of Enteric Glial Cells
Source: Mar Drugs. 2019 Jul 23;17(7):429. doi: 10.3390/md17070429 (PMC6669610; doi:10.3390/md17070429)
Supplement: Supplementary file 1 [file marinedrugs-17-00429-s001.pdf]

marine drugs

Supplementary Materials:

Table S1. Summary of primers used for qPCR analysis. F : forward primer, R : reverse primer.

Gene		Sequence (5'to 3')
ACTB	F	GATCAAGATCATTGCTCCTCCTGA
	R	ACGCAGCTCAGTAACAGTCC
ADH5	F	CGAACCAGGTGATCAGGTGTAA
	R	GTATAGGCATCGGTGTGGCA
ADORA2B	F	GCTTCTGCACGGACTTTCAC
	R	AACCTTTATACCTGAGCGGGAC
ATG10	F	GGCAAGCTTTTTAGATGGGAGAC
	R	GTTGCCCAAGTATTGGATGCTC
BAX	F	ACACCTGAGCTGACCTTGGA
	R	AGTTCATCGCCAATTCGCCT
BCL2	F	CTGGTGGACAACATCGCTCT
	R	TCCACAAAGGCATCCCAGC
BDNF	F	TACCTGGATGCCGCAAACAT
	R	TGGCCTTTTGATACCGGGAC
BNIP3	F	AAAACAGCACTCTGTCTGAGGAA
	R	TTTCTGGCCGACTTGACCAAT
CAPN1	F	TCATTTCCAGCTGTGGCAGT
	R	AGCTTTCCATCCTTGGTGGG
CAPN2	F	GAGGCATTGCCGAGTGGTAT
	R	GACGGAGTACGCATGTCCTT
CAST	F	TGGCACTGAGAGGAGAGACA
	R	TGGATCAGGCTGTCTTTGTCC
CASP3	F	GGAGCTTGGAACGCGAAGA
	R	TACACAAGCCCATTTCAGGGT
CAT	F	CCGCCTTTTTGCTTACCCAG
	R	GAGCACGGTAGGGACAGTTC
CCNA2	F	AGTGCCGCTGTCTCTTTACC
	R	GGGGTGATTCAAAACTACCATCC
CRB1	F	CGTGTACTTGGCCCTTTTGC
	R	TCAGGATAGAAGGGGTGGGG
CDK1	F	GAACAGAGAGGGTCCGTTGTAA
	R	GTACTGGGCACTCCTTCTTCC
CDK2	F	CTTTGCCGAAATGGTGACCC
	R	TAACTCCTGGCCAAACCACC
CYCS	F	CTTGGGCTAGAGAGCGGGA
	R	CCCATTTTAAATTCGGTCCGGG

FOS	F	CCCTGTCAACACACAGGACTT
	R	GCTGTCACCGTGGGGATAAA
GJA1	F	AGGTCTGAGAGCCTGAACTCT
	R	CTGGGCACCTCTCTTTCACTTA
EGF	F	AGAACGATGTCAGCACCGAG
	R	CTGCTTCCGAGTCCTGTAGTAG
MAPK3	F	AACCCAAACAAGCGCATCAC
	R	AGCCACTGGTTCATCTGTCG
MAPK1	F	CTTAAATTGGTCAGGACAAGGGC
	R	CGGCTCAAAGGAGTCAAGAGT
FZD4	F	CACCACTGTCCAACCTCCTC
	R	TCTACCCTACACAGGGCTCC
GABARAP	F	CTGTACCAGGAACACCATGAAGA
	R	GCGCCACCTCTCTTTGTAGA
GAPDH	F	AGTGCCAGCCTCGTCTCATA
	R	GGTAACCAGGCGTCCGATAC
GCL	F	TGTCGCTGGGGAGTGATTTC
	R	GATTGTTCTTCAGGGGCTCCA
GDNF	F	CACCAGATAAACAAGCGGCG
	R	TCGTAGCCCAAACCCAAGTC
GFAP	F	AACGTTAAGCTAGCCCTGGAC
	R	CAGGAATGGTGATGCGGTTTT
GFRA1	F	CCAGCCAGAGTCAAGGTCTG
	R	TGACTGTGCCAATCAGTCCC
GPX1	F	GGTAGGTCCAGACGGTGTTC
	R	ATCGGGTTCGATGTCGATGG
GSS	F	TGTGCACCGACATGTTCTCA
	R	CTCGTTCTCTATGGCACGCT
IL1R1	F	CCAAGACCTACGGAGAGGGA
	R	ACCTCGATGGTATCTTCCCCA
IL6	F	GAGTGGCTAAGGACCAAGACC
	R	TAGCACACTAGGTTTGCCGAG
MAPK8	F	TGCTGGTGATAGATGCGTCC
	R	CCAGACGTTGATGTACGGGT
LC3B	F	AAGAGTGGAAGATGTCCGGC
	R	TGCAGGCGCCTTCTAATTATCT
LPA1	F	CCGACTCACGAGTTGCTTCT
	R	CTGTGAACTGGGGCTGTGAA
MAPK14	F	CCAGCTTCAGCAGATAATGCG
	R	TGTAGTTTCTTGCCTCATGGCT

CCL2	F	CTGTAGCATCCACGTGCTGT
	R	AGTTCTCCAGCCGACTCATTG
MTOR	F	CACCCATCCAACCTGATGCT
	R	ATCGAGACCGGTAACCTCCA
MYD88	F	CCTGTCTCCAGGTGTCCAAC
	R	GGGTCCAGAACCAGGACTTG
NFKB1	F	TGTCAACAGATGGCCCATACC
	R	TTTGCAGGCCCCACATAGTT
NGF	F	CATCGCTCTCCTTCACAGAGTT
	R	GACATTACGCTATGCACCTCAG
NGFR	F	GCTGCTGCTGATTCTAGGGAT
	R	GAGAATGTAACACTGTCCAGGCA
NFE2L2	F	CATTTGTAGATGACCATGAGTCGC
	R	CGTATTAAGACACTGTAACTCGGGA
P2Y4	F	TAAGGAAGCTAGGGGGCCAT
	R	CGGAAGAGGAACAGCCAGAG
PRKACA	F	ATCTATCAGAGAAAGGTGGAAGCTC
	R	ACAAGCACACCCCTAAAACTCA
RAGE	F	TGAGGTAGGGCATGAGGATGA
	R	ATCACCGGTTTCTGTGACCCT
REDD1	F	GCTAGCTGCGGCTTCTGT
	R	GGAGGACGAGAAACGATCCC
RELA	F	GATAACCGTGCCCCCAACA
	R	AGCCAGGTCCCGTGAAATAC
RHOA	F	CGGGAAGCAGGTAGAGTTGG
	R	TGTCTGGGTAGGAGAGAGGC
S100B	F	TTCCTGGAGGAAATCAAAGAGCA
	R	GAAGTCACACTCCCCATCCC
SOD1	F	TGGGGACAATACACAAGGCTG
	R	TCCAACATGCCTCTCTTCATCC
SOD2	F	GCCTCAGCAATGTTGTGTCG
	R	ATTGTTCACGTAGGTCGCGT
SOX10	F	CAAGCTCTGGAGGTTGCTGA
	R	GGGCTGCCTTCCCATTCTT
TGFB1	F	GCTGAACCAAGGAGACGGAATA
	R	CCTCGACGTTTGGGACTGAT
TLR4	F	ACTGGGTGAGAAACGAGCTG
	R	CAGCAATGGCTACACCAGGA
TRPV1	F	AGGGAGATCCATGAACCCGA
	R	CATGTCATGACGGTTAGGGGT

VIM	F	GCTGCGAGAAAAATTGCAGGA
	R	GGTCAAGACGTGCCAGAGAA

Table S2. Relative gene expression in EGCs after 24 hrs exposure to PTX2, YTX and AZA1. The gene expression was carried out by RT-qPCR. Values are normalized to the reference gene GAPDH and presented as mean ± SEM. Three independent experiments were performed.

Biological function	Gene		Control	PTX2 1 nM	PTX2 2 nM	PTX2 4 nM	YTX 1 nM	YTX 2 nM	YTX 4 nM	AZA1 0.38 nM	AZA1 0.75 nM	AZA1 1.5 nM
Viability	ATG10	Mean	5.85	7.41	6.07	4.76	6.76	5.04	6.56	6.80	7.19	6.65
		SEM	0.73	1.24	0.56	0.54	0.49	0.88	0.52	0.55	0.32	1.64
	BAX	Mean	0.96	1.04	1.07	0.84	0.95	1.04	0.91	1.00	1.07	1.00
		SEM	0.12	0.10	0.01	0.03	0.02	0.06	0.02	0.03	0.12	0.07
	CAPN1	Mean	0.83	0.99	0.96	0.83	0.89	0.81	0.81	0.90	0.71	0.77
		SEM	0.08	0.05	0.11	0.07	0.08	0.13	0.06	0.04	0.11	0.05
	CAPN2	Mean	0.96	1.20	1.16	1.06	1.09	1.10	0.99	1.09	1.10	1.21
		SEM	0.03	0.08	0.15	0.06	0.04	0.05	0.09	0.05	0.09	0.05
	LC3B	Mean	1.07	1.07	1.12	0.82	1.03	1.02	1.09	1.00	1.19	1.22
		SEM	0.07	0.16	0.24	0.21	0.06	0.10	0.17	0.15	0.04	0.04
	MTOR	Mean	4.80	5.58	5.25	4.06	4.73	4.55	4.27	5.24	4.44	4.66
		SEM	0.50	0.64	0.22	0.49	0.13	0.48	0.05	0.46	0.25	0.14
	REDD1	Mean	3.94	4.31	4.60	3.03	4.59	4.56	4.32	4.32	4.42	4.45
		SEM	0.36	0.31	0.53	0.50	0.28	0.26	0.27	0.38	0.37	0.29
Inflammation	IL6	Mean	13.26	12.57	18.70	65.14	13.09	20.21	3.35	11.32	15.89	14.75
		SEM	2.74	1.90	5.58	44.25	4.85	10.58	0.48	3.44	10.62	5.37
	MAPK1	Mean	3.43	4.11	4.28	3.61	3.49	3.29	3.21	3.85	3.32	3.51
		SEM	0.53	0.16	0.26	0.24	0.44	0.24	0.28	0.73	0.51	0.51
	MAPK3	Mean	1.05	1.11	1.14	0.86	1.17	0.96	0.96	1.12	1.16	1.07
		SEM	0.03	0.05	0.11	0.09	0.03	0.06	0.03	0.02	0.05	0.07
	MAPK8	Mean	0.57	0.80	0.82	0.74	0.77	0.66	0.77	0.83	0.75	0.78
		SEM	0.08	0.07	0.08	0.04	0.03	0.05	0.03	0.03	0.04	0.09
	MAPK14	Mean	4.16	5.10	4.87	3.76	4.69	3.83	4.07	5.70	4.97	5.00

		SEM	0.38	0.82	0.23	0.37	0.20	0.42	0.20	0.94	0.83	0.11
	MCP1	Mean	0.49	0.69	0.53	0.46	0.70	1.07	0.90	0.87	1.14	1.22
		SEM	0.19	0.04	0.01	0.08	0.05	0.15	0.12	0.19	0.01	0.14
	NFKB1	Mean	4.15	4.60	5.27	5.01	5.27	3.37	3.85	4.16	4.23	2.98
		SEM	0.89	0.25	0.44	0.30	0.75	0.60	0.22	0.18	1.11	0.09
	RELA	Mean	1.07	1.05	1.05	0.92	0.94	0.99	1.05	0.69	1.04	1.03
		SEM	0.02	0.02	0.14	0.13	0.05	0.08	0.15	0.04	0.08	0.08
Oxidative stress	ADH5	Mean	5.08	5.50	5.91	3.91	5.14	4.51	4.88	5.57	5.14	5.50
		SEM	0.08	0.52	0.06	0.68	0.31	0.14	0.10	0.29	0.20	0.24
	CAST	Mean	1.03	1.18	1.11	1.10	1.04	1.11	0.98	1.14	1.12	1.15
		SEM	0.06	0.06	0.07	0.11	0.03	0.11	0.04	0.06	0.03	0.07
	CRB1	Mean	3.38	4.11	3.59	3.77	3.94	4.44	3.52	4.91	4.03	5.06
		SEM	0.21	0.41	0.11	0.48	0.17	0.24	0.16	1.11	0.07	0.50
	CYCS	Mean	1.10	1.23	1.17	1.04	1.06	0.99	0.98	1.24	1.19	1.20
		SEM	0.03	0.03	0.01	0.07	0.03	0.04	0.05	0.09	0.05	0.05
	GCL	Mean	0.70	0.60	0.96	0.85	0.69	0.82	0.82	0.95	0.85	1.00
		SEM	0.07	0.08	0.07	0.10	0.09	0.11	0.08	0.18	0.02	0.09
	GPX1	Mean	1.07	1.10	1.23	1.05	1.05	0.77	0.73	0.94	0.90	0.90
		SEM	0.12	0.09	0.22	0.07	0.15	0.11	0.12	0.31	0.11	0.02
	GSS	Mean	3.88	5.24	3.71	2.92	4.56	3.69	4.16	4.44	4.24	3.09
		SEM	0.39	0.71	0.09	0.53	0.49	0.65	0.31	0.30	0.63	0.21
	SOD1	Mean	0.91	1.06	0.92	0.86	0.95	0.94	0.91	0.95	1.01	1.07
		SEM	0.04	0.10	0.08	0.05	0.02	0.07	0.01	0.03	0.02	0.10
	SOD2	Mean	10.81	11.81	11.04	11.20	10.56	12.22	13.72	11.14	11.46	14.24
		SEM	1.02	1.05	1.10	2.08	0.92	1.07	0.74	1.13	1.48	1.74

Gliomediator	EGF	Mean	6.45	4.91	6.54	5.01	5.06	5.33	3.42	5.87	4.77	5.41
		SEM	0.56	0.96	1.27	0.11	0.07	1.31	0.71	1.09	0.90	0.66
	NGF	Mean	28.36	18.16	16.50	16.14	19.84	21.59	17.28	32.12	20.41	23.27
		SEM	6.85	1.71	0.87	1.25	4.42	0.64	6.60	5.46	1.48	10.17
	S100B	Mean	0.82	0.96	0.91	0.75	0.90	0.97	1.01	0.96	1.05	1.11
		SEM	0.02	0.14	0.16	0.02	0.08	0.07	0.05	0.01	0.04	0.12
	TGFB1	Mean	0.80	0.92	0.93	1.03	0.88	0.80	0.79	0.91	0.88	0.81
		SEM	0.01	0.13	0.15	0.10	0.06	0.06	0.09	0.03	0.03	0.08
Channel and receptor	ADORA2B	Mean	63.06	19.09	43.04	38.87	47.20	102.82	35.33	154.39	54.80	55.36
		SEM	29.88	16.67	20.26	23.51	37.44	100.01	12.79	100.00	18.13	26.93
	P2Y4	Mean	18.49	15.20	15.39	21.42	17.11	13.89	15.21	17.23	16.02	13.49
		SEM	2.16	4.79	4.14	4.65	0.39	1.02	1.52	2.03	1.08	3.20
	NGFR	Mean	0.73	0.91	0.89	0.97	0.86	0.80	0.94	0.84	1.00	0.97
		SEM	0.02	0.08	0.16	0.13	0.06	0.04	0.06	0.07	0.05	0.05
	RAGE	Mean	14.49	9.67	17.51	14.19	12.58	10.21	10.92	12.85	13.52	10.71
		SEM	2.03	2.63	2.65	2.57	1.47	2.60	1.99	1.68	2.89	3.36
	TRPV1	Mean	5.01	1.55	5.99	5.94	3.99	3.99	3.54	3.80	3.66	2.77
		SEM	3.17	0.62	2.53	2.54	1.06	0.27	0.40	1.52	0.32	0.43
Morphology	ACTB	Mean	1.13	1.18	1.06	0.86	1.07	0.88	0.72	1.16	0.95	0.88
		SEM	0.01	0.02	0.01	0.05	0.02	0.03	0.00	0.16	0.13	0.04
	PRKACA	Mean	0.89	1.15	1.14	1.02	0.97	0.84	0.71	0.95	1.08	0.87
		SEM	0.06	0.18	0.13	0.25	0.10	0.08	0.08	0.07	0.06	0.09
	SOX10	Mean	0.82	0.98	0.98	0.85	0.84	0.76	0.86	0.83	0.90	0.79
		SEM	0.06	0.10	0.10	0.02	0.01	0.02	0.11	0.04	0.04	0.06
	VIM	Mean	0.95	1.03	1.00	0.95	0.86	0.76	0.77	0.95	0.80	0.76

		SEM	0.10	0.06	0.07	0.07	0.07	0.04	0.10	0.03	0.04	0.10
